# Bioinformatics-based screening of key genes for transformation of liver cirrhosis to hepatocellular carcinoma

**DOI:** 10.1186/s12967-020-02229-8

**Published:** 2020-01-30

**Authors:** Chen Hao Jiang, Xin Yuan, Jiang Fen Li, Yu Fang Xie, An Zhi Zhang, Xue Li Wang, Lan Yang, Chun Xia Liu, Wei Hua Liang, Li Juan Pang, Hong Zou, Xiao Bin Cui, Xi Hua Shen, Yan Qi, Jin Fang Jiang, Wen Yi Gu, Feng Li, Jian Ming Hu

**Affiliations:** 1 Department of Pathology and Key Laboratory for Xinjiang Endemic and Ethnic Diseases (Ministry of Education), Shihezi University School of Medicine, Xinjiang, 832002 China; 2 Department of Pathology, The First Affiliated Hospital, Shihezi University School of Medicine, Xinjiang, 832002 China; 3 Department of Pathology, Beijing Chaoyang Hospital, Capital Medical University, Beijing, China; 4 Australian Institute of Bioengineering and Nanotechnology, University of Queensland, Brisbane, QLD 4072 Australia

**Keywords:** Hepatocellular carcinoma, Liver cirrhosis, Bioinformatics, CDKN3, Microarray, Protein–protein interactions, Molecular markers

## Abstract

**Background:**

Hepatocellular carcinoma (HCC) is the most common type of liver tumour, and is closely related to liver cirrhosis. Previous studies have focussed on the pathogenesis of liver cirrhosis developing into HCC, but the molecular mechanism remains unclear. The aims of the present study were to identify key genes related to the transformation of cirrhosis into HCC, and explore the associated molecular mechanisms.

**Methods:**

GSE89377, GSE17548, GSE63898 and GSE54236 mRNA microarray datasets from Gene Expression Omnibus (GEO) were analysed to obtain differentially expressed genes (DEGs) between HCC and liver cirrhosis tissues, and network analysis of protein–protein interactions (PPIs) was carried out. String and Cytoscape were used to analyse modules and identify hub genes, Kaplan–Meier Plotter and Oncomine databases were used to explore relationships between hub genes and disease occurrence, development and prognosis of HCC, and the molecular mechanism of the main hub gene was probed using Kyoto Encyclopedia of Genes and Genomes(KEGG) pathway analysis.

**Results:**

In total, 58 DEGs were obtained, of which 12 and 46 were up- and down-regulated, respectively. Three hub genes (CDKN3, CYP2C9 and LCAT) were identified and associated prognostic information was obtained. CDKN3 may be correlated with the occurrence, invasion, and recurrence of HCC. Genes closely related to changes in the CDKN3 hub gene were screened, and Kyoto Encyclopedia of Genes and Genomes (KEGGs) pathway analysis identified numerous cell cycle-related genes.

**Conclusion:**

CDKN3 may affect the transformation of liver cirrhosis into HCC, and represents a new candidate molecular marker of the occurrence and progression of HCC.

## Background

Hepatocellular carcinoma (HCC) is the fifth most common malignant tumour worldwide, and the second deadliest [1]. Risk factors for HCC include hepatitis B and C infection, liver cirrhosis and alcohol intake [2]. In many liver diseases, there are no significant differences in the incidence of liver cancer between cirrhotic and non-cirrhotic states. However, compared with the non-cirrhotic state, the incidence of HCC in the cirrhotic state is significantly increased (2.79–45.00-fold) [3]. Current molecular markers of liver cancer, such as α-fetal protein [4], decarboxylation of thrombin [5] and insulin-like growth factor [6] cannot clearly distinguish between liver cancer and liver cirrhosis. Additionally, treatment of early hepatocellular carcinoma can improve the survival rate of patients [7]. Therefore, it is very important to understand the mechanism by which liver cirrhosis develops into liver cancer, and explore the molecular characteristics of HCC occurrence, development, and poor prognosis to provide new strategies for the effective prevention, diagnosis and treatment of HCC.

Microarray and bioinformatics approaches have been widely used to screen genetic changes at the genome level. Herein, we analysed four mRNA microarray datasets from Gene Expression Omnibus (GEO) to obtain differentially expressed genes (DEGs) between HCC and liver cirrhosis tissues. Subsequently, protein–protein interaction (PPI) network analysis was carried out to explore relationships between different genes, hub genes were screened using the MCODE plug-in, and relationships between hub genes and prognosis were analysed by Kaplan–Meier Plotter. Combined with ONCOMINE database analysis, CDKN3 was identified as a key hub gene closely correlated with the progression of HCC. cBioPortal was used to identify genes closely related to changes in CDKN3, and Kyoto Encyclopedia of Genes and Genomes (KEGG pathway) analysis was used to analyse the mechanism by which CDKN3 may affect the occurrence and development of HCC. The findings provide new candidate molecular markers for studying the occurrence and development of HCC.

## Materials and methods

### Microarray data

Genes were screened using the GEO (http://www.ncbi.nlm.nih.gov/geo) database, specifically the GSE89377 series on the GPL6947 platform (Illumina HumanHT12 V3.0 expression beadchip), the GSE17548 series [8] on the GPL570 platform (Affymetrix Human Genome U133 Plus 2.0 Array), the GSE63898 series [9] on the GPL13667 platform (Affymetrix Human Genome U219 Array), and the GSE54236 series [10] on the GPL6480 platform (Agilent-014850 Whole Human Genome Microarray 4x44K G4112F), the basic clinical info of the patients selected is showed in Additional file 1: Table S1. According to the annotation information in each platform, probes were converted into corresponding gene symbols. The GSE89377 dataset contained 40 HCC tissue samples and 12 liver cirrhosis samples, the GSE17548 dataset contained 18 HCC tissue samples and 19 liver cirrhosis samples, the GSE63898 dataset contained 228 HCC tissue samples and 168 liver cirrhosis samples, and the GSE54236 dataset contained 81 HCC tissues samples and 80 liver cirrhosis samples.

### Identification of DEGs

GEO2R (https://www.ncbi.nlm.nih.gov/geo/geo2r/) was used to identify DEGs between HCC and liver cirrhosis samples. GEO2R is an interactive network tool that allows users to compare two or more datasets in the GEO series to identify DEGs under experimental conditions [11]. Genes without a corresponding gene symbol, and genes with more than one probe set are separately removed, and fold change (FC) > 2 and adjusted *p* < 0.05 are the threshold criteria for statistical significance. In order to identify significant DEGs, the Venn online tool (http://bioinformatics.psb.ugent.be/webtools/Venn/) was used to draw a Venn map, and overlapping DEGs were retained for further analysis.

### Construction and module analysis of the PPI network

The String online database (http://string-db.org Version:11.0) [12] was used to build a PPI network. Analysis of functional interactions between proteins can be helpful for understanding mechanisms related to disease occurrence and development. Herein, a comprehensive Gt score > 0.4 was considered statistically significant. The molecular composite detection plug-in MCODE within Cytoscape [13] was used to cluster the resulting network in order to reveal closely connected regions. The most important module in the PPI network was identified by MCODE. The selection criteria were MCODE score > 5, cut-off value = 2, node cut-off value = 0.2, maximum depth = 100, and k-score = 2.

### Hub gene analysis

Correlations between hub genes was analysed which came from TCGA using cBioPortal (http://www.cbioportal) [14]. The online database the Wurmbach liver dataset [15] which comes from Oncomine (http://www.oncomine.com) [16] was used to analyse expression levels of key genes under normal, cirrhotic, hepatocellular and hepatocyte dysplasia conditions. Overall survival related to hub genes was analysed using the Kaplan–Meier curve feature of Kaplan–Meier Plotter which includes 364 patients [17]. With the Wurmbach liver dataset, Oncomine was used to analyse differential expression of core genes in different studies, and to analyse the relationships between expression level and liver cancer tumour grade, hepatitis virus infection status, satellites, and vascular invasion, and to identify core genes most closely related to the development of HCC [16]. DEGs closely related to core genes were further screened by cBioPortal [18], and KEGG pathway analysis of genes related to DEGs was performed using DAVID [19]. The resulting pathway map was drawn using KEGG [20]. The relevant positions of corresponding genes in the pathway were coloured red.

## Results

### Identification and analysis of DEGs in liver cancer tissues

**Fig. 1:**
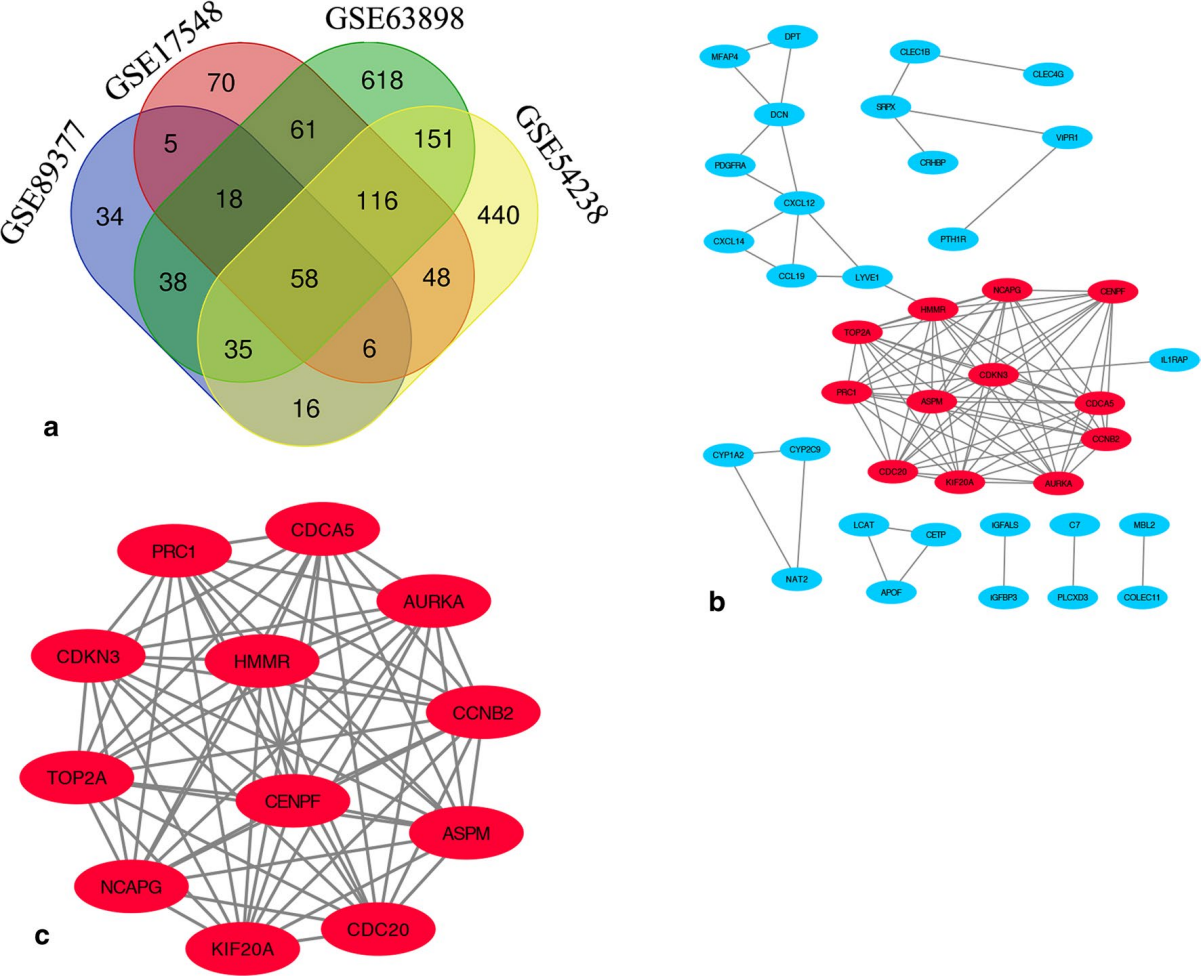
Venn diagram and PPI network analyses, showing the most significant module related to DEGs. **a** DEGs were identified from GSE17548, GSE63898, GSE54238 and GSE89377 gene expression profiling datasets based on fold change > 2 and adjusted *p* value < 0.05. The four datasets share 58 overlapping DEGs. **b** PPI network constructed using 58 DEGs. Up- and down-regulated genes were marked in red and blue, respectively. **c** The most significant module of the PPI network includes 12 nodes and 66 edges

DEGs identified in the four microarray datasets (382 in GSE17548, 870 in GSE54236, 1094 in GSE63898, and 210 in GSE89377) were screened after the chip results were normalised. As shown in the Venn map, 58 genes overlapped in the four datasets (Fig. 1a, Additional file 2: Table S2), comprising 12 and 46 up- and down-regulated genes. STRING database screening and PPI network construction were performed, and visualization was carried out using Cytoscape software (Fig. 1b). The PPI network was constructed and significant modules were identified, with 93 edges and 39 nodes in the PPI network. MCODE plugin used to find the top hub genes, the most closely connected module was identified (Fig. 1c); it includes 13 nodes and 66 edges, and genes in this region were upregulated in HCC tissues.

### Selection and analysis of hub genes

**Fig. 2:**
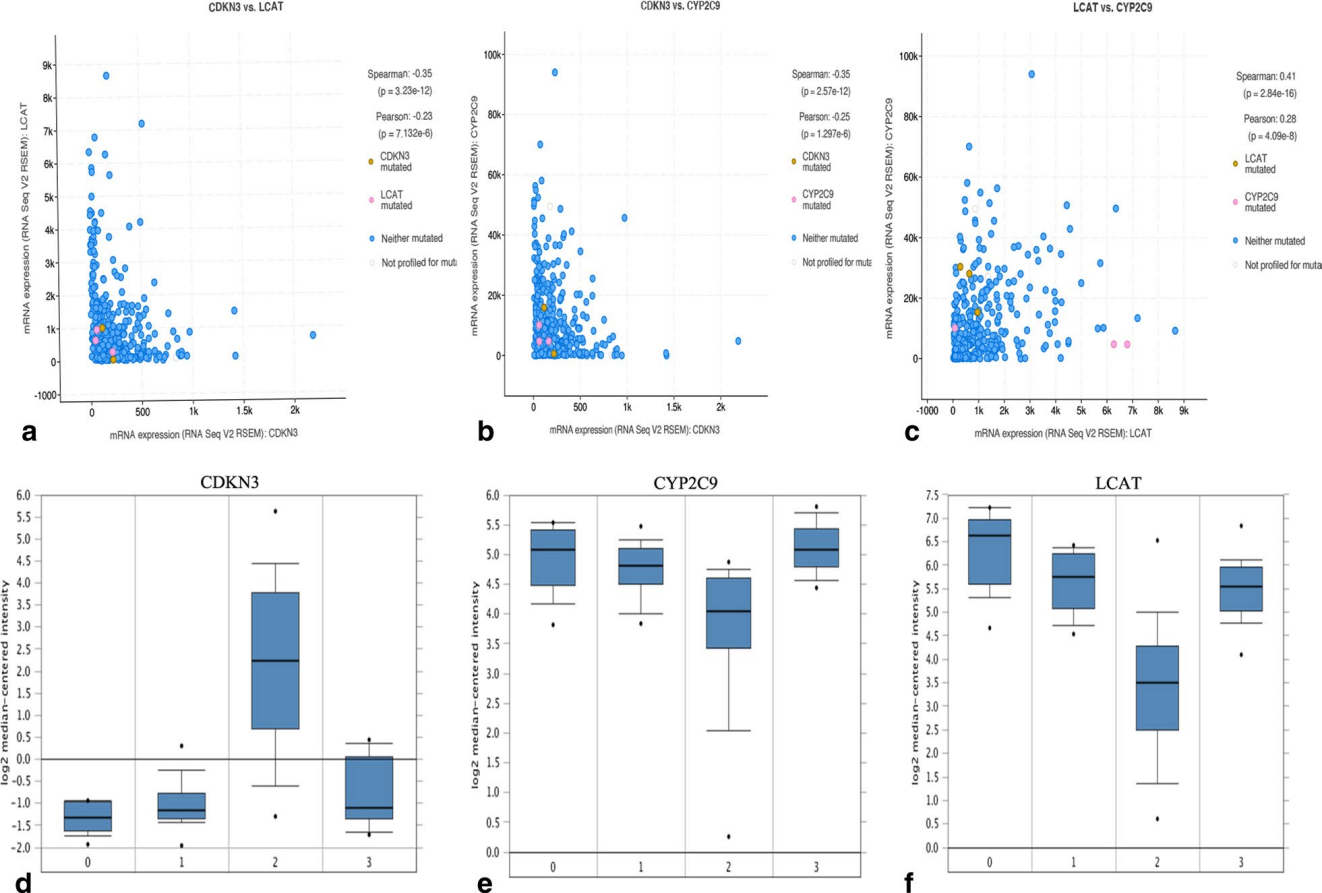
Relationships between CDKN3, CYP2C9 and LCAT, and their expression in different types of liver tissues. **a**–**c** Correlations between CDKN3, CYP2C9 and LCAT in liver hepatocellular carcinoma, p < 0.05 was considered statistically significant. **d**–**f** Expression of CDKN3, CYP2C9 and LCAT under normal, cirrhotic, hepatocellular carcinoma and hepatic dysplasia conditions in the Wurmbach liver dataset

**Fig. 3:**
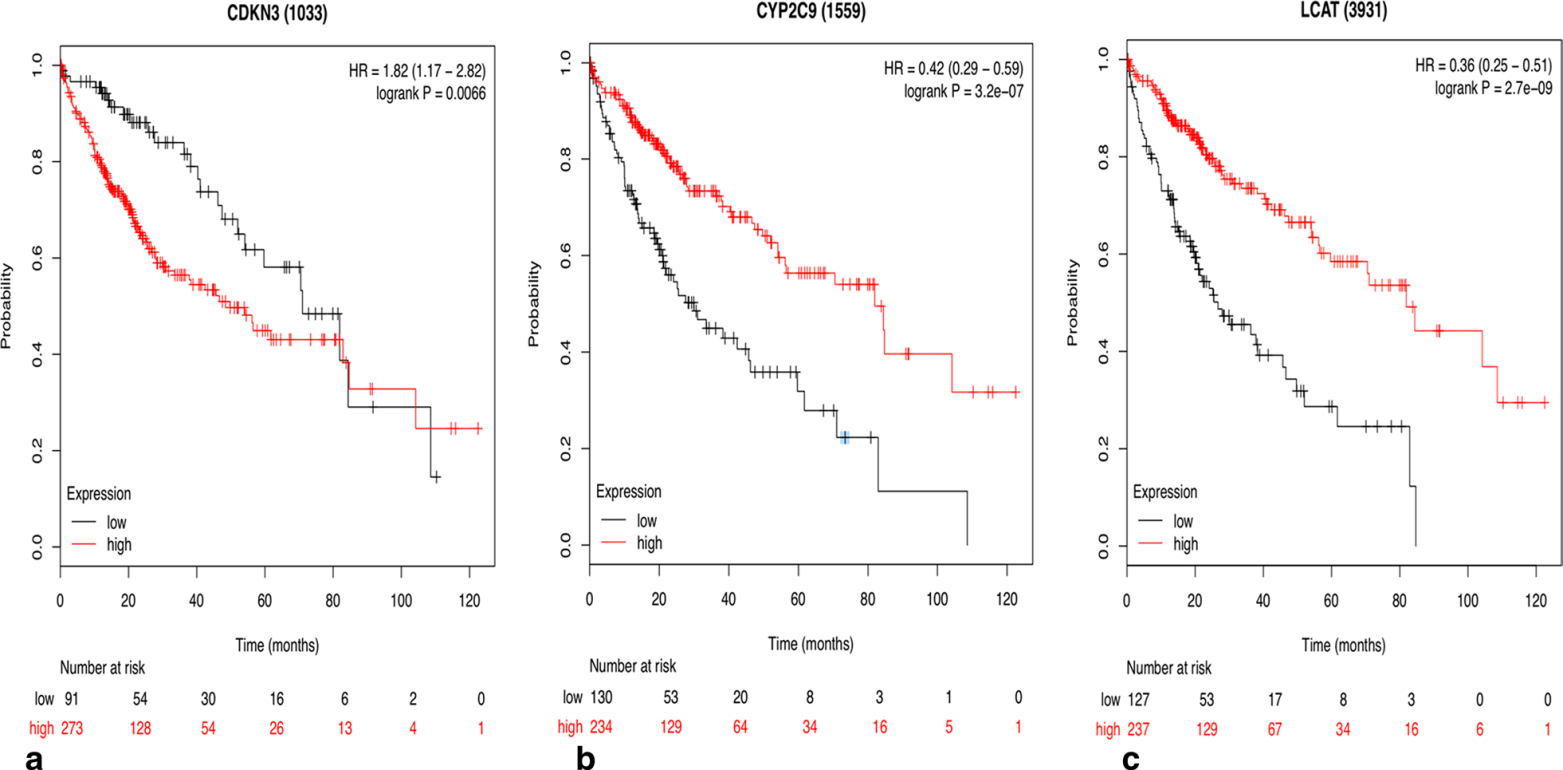
Overall survival analyses of hub genes (CDKN3, CYP2C9 and LCAT) performed using the Kaplan–Meier Plotter online platform, p < 0.05 was considered statistically significant

**Fig. 4:**
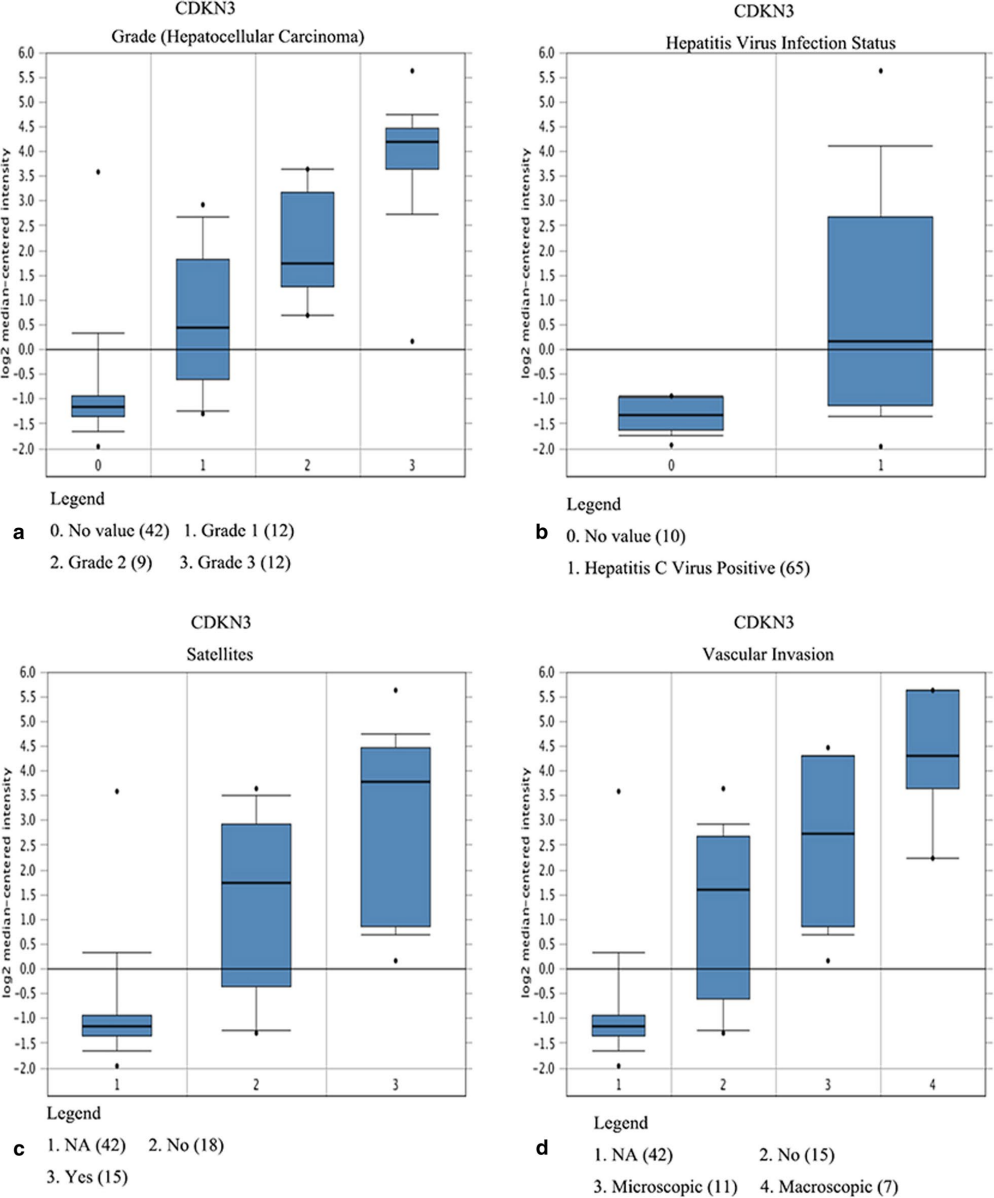
Association between the expression of CDKN3 and tumour grade (**a**), hepatitis virus infection status (**b**), satellites (**c**), and vascular invasion (**d**) in the Wurmbach liver dataset

Three hub genes (seed genes) were identified by Cytoscape software, for which the names, abbreviations, and functions are listed in Table 1. Furthermore, there was a significant correlation among the three hub genes (*p* < 0.05) according to the cBioPortal database (Fig. 2a–c), demonstrating a specific interaction between hub genes. Additionally, the Oncomine database was used to analyse the expression of the hub gene under normal, cirrhosis, hepatocellular carcinoma and hepatocyte dysplasia conditions(Fig. 2d–f). There were no significant differences in CDKN3 between normal, cirrhotic and hepatocyte dysplasia, but expression was increased significantly in HCC (Fig. 2d). Furthermore, there were no significant differences between CYP2C9 and LCAT in normal, liver cirrhosis and hepatocyte dysplasia, but levels were decreased in HCC (Fig. 2e–f). This indicates that the hub gene plays an important role in the development of cirrhosis into HCC. However, it does not play an important role in normal, liver cirrhosis and hepatocyte dysplasia. Survival-related hub genes were analysed using the Kaplan–Meier curve feature within the Kaplan–Meier Plotter database which included 364 cases of hepatocellular carcinoma (Fig. 3). We noted that HCC patients with elevated CDKN3 levels were associated with a decrease in overall survival (*p *< 0.05) (Fig. 3a). And, patients with high expression of CYP2C9 or LCAT have a higher survival rate(*p *< 0.05) (Fig. 3b–c). We also noted that in the Wurmbach liver dataset, the mRNA expression level of CDKN3 was associated with tumour grade, hepatitis virus infection status, and satellite and vascular invasion (Fig. 4), but CYP2C9 and LCAT were not associated with tumour development (Additional file 3: Fig. S1 and Additional file 4: Fig. S2). These results suggest that CDKN3 may be a key gene in the transformation of liver cirrhosis to liver cancer, and is closely related to the occurrence and development of HCC.

**Table 1:** Functional roles of hub genes with a degree ≥ 10

Gene symbol	Full name	Function
CDKN3	Cyclin-dependent kinase inhibitor 3	The protein was identified as a cyclin-dependent kinase inhibitor, and has been shown to interact with and dephosphorylate CDK2 kinase, thereby preventing the activation of CDK2 kinase. This gene was reported to be deleted, mutated, or overexpressed in several kinds of cancers
CYP2C9	Cytochrome P450 family 2 subfamily C member 9	This gene encodes a member of the cytochrome P450 superfamily of enzymes. These monooxygenases catalyse many reactions involved in drug metabolism and the synthesis of cholesterol, steroids and other lipids
LCAT	Lecithin-cholesterol acyltransferase	This gene encodes the extracellular cholesterol esterifying enzyme lecithin-cholesterol acyltransferase. Esterification of cholesterol is required for cholesterol transport

**Fig. 5:**
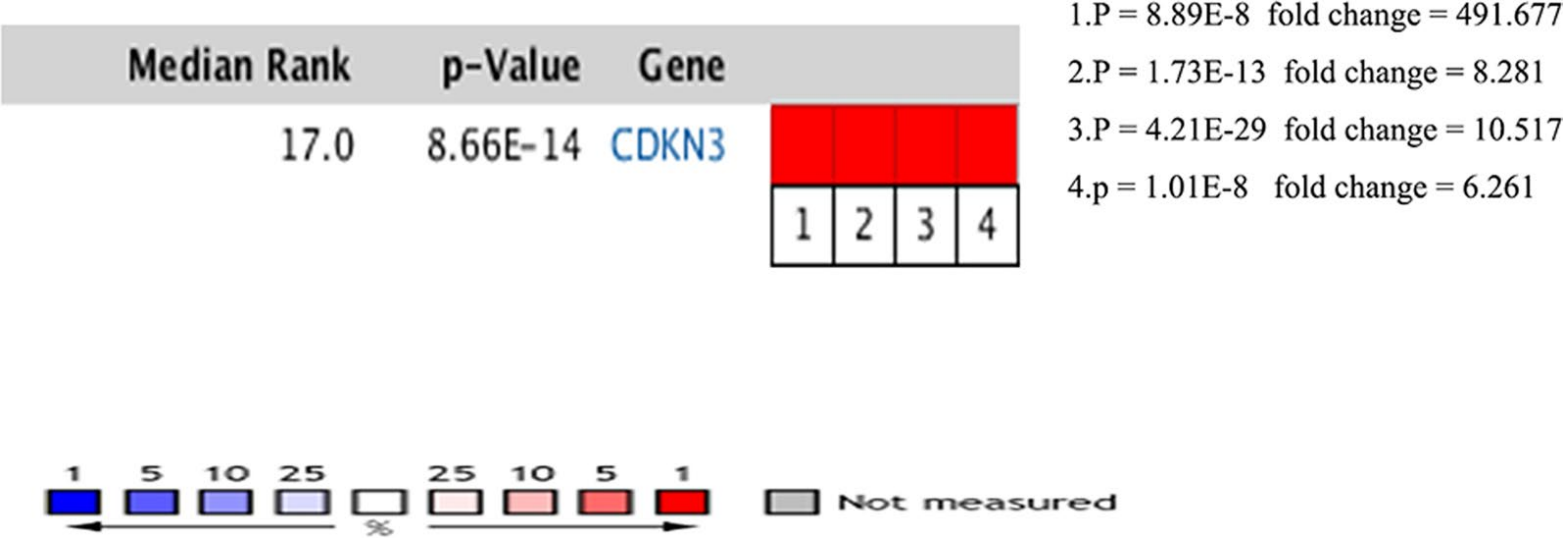
Oncomine analysis of CDKN3 expression in cancer vs. normal tissues. Heatmaps represent CDKN3 gene expression in carcinoma samples vs. normal tissues. 1. Adrenal cortex carcinoma vs. normal tissues (Giordano Am J Pathol, 2003 [45]); 2. Hepatocellular carcinoma vs. normal tissues (Wurmbach [17]); 3. Squamous cell lung carcinoma vs. normal tissues (Hou PLoS One, 2010 [46]); 4. Adrenal cortex carcinoma vs. normal tissue (Giordano Am J Pathol, 2003)

Meanwhile, Oncomine analysis showed that CDKN3 was significantly up-regulated in anaplastic oligodendrocytoma, leukaemia, hepatocellular carcinoma and sarcoma (Fig. 5).

### Screening and analysis of genes related to CDKN3 expression in HCC

**Fig. 6:**
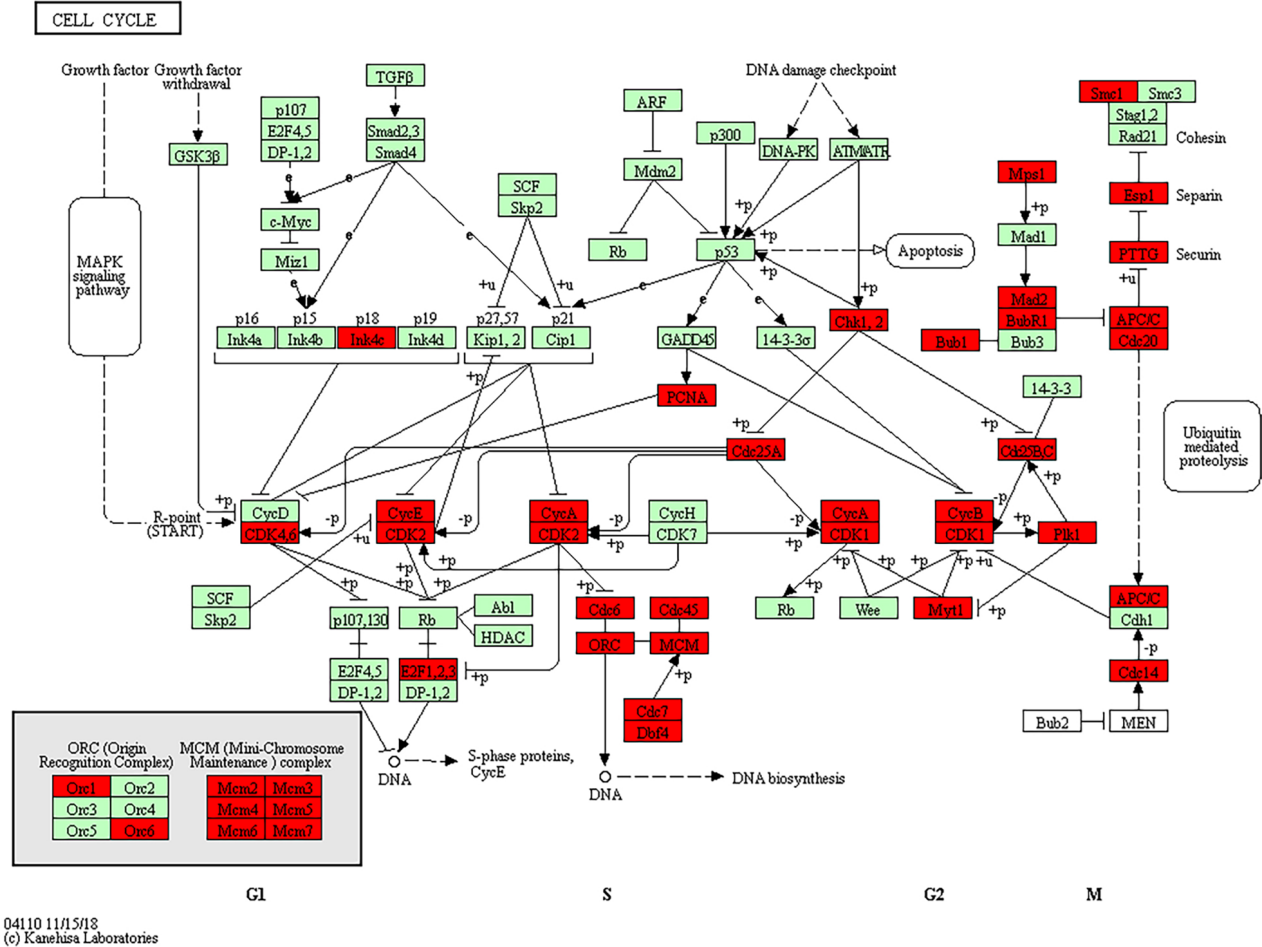
Positional relationships of CDKN3-associated genes in the cell cycle. The CDKN3-related genes in the cell cycle pathway are brought into the website related to the KEGG pathway to generate a road map, and the relevant positions of the corresponding genes in the pathway are marked with red circles, most of which are related to the S phase and G2/M phase of the cell cycle

In order to understand the expression of CDKN3 in different tumours, Oncomine analysis was performed, and the results showed that CDKN3 was significantly elevated in anaplastic oligodendrocytoma, leukaemia, hepatocellular carcinoma and sarcoma (Fig. 5). In order to further analyse the potential mechanism of the influence of CDKN3 on HCC, the co-expression genes of CDKN3 in TCGA HCC transcriptome data were screened by cBioProtal data analysis platform, Pearson and Spearman scores are more than 0.3. The top 20 genes are listed in Table 2. Correlations with expression levels were evaluated by Pearson and Spearman scores. KEGG pathways related to the top 500 related genes were analysed by DAVID online software, and the top 8 hits are shown in Table 3. The results showed that changes in CDKN3 expression mainly affected pathways related to HCC, such as the cell cycle, DNA replication, oocyte meiosis and mismatch repair. We observed that most genes with strong correlations to CDKN3 expression were involved in cell cycle regulation, and these genes were used to generate a KEGG pathway map (Fig. 6), which relevant positions of the corresponding genes in the pathway are coloured red. The results showed that ORC, MCM, Chk1 and 2, and some other genes occupy important positions, mainly located in the S or G2/M phases of the cell cycle, and they regulate the G1 phase of the gene. Thus, CDKN3 mainly inhibits the transition from the G 1 to the S phase, and plays an important role in inhibiting cancer progression.

**Table 2:** cBioPortal analysis of the 20 genes most closely related to CDKN3

Gene	Cytoband	Spearman’s correlation	p-value	q-value
SPC25	2q24.3	0.86085851	6.68E−111	1.34E−106
CDK1	10q21.2	0.85877853	8.53E−110	8.57E−106
CCNB2	15q22.2	0.85556873	4.02E−108	2.69E−104
NDC80	18p11.32	0.85377317	3.33E−107	1.67E−103
DLGAP5	14q22.3	0.85123904	6.27E−106	2.52E−102
CCNB1	5q13.2	0.84636895	1.52E−103	5.10E−100
NCAPG	4p15.31	0.84493736	7.38E−103	2.12E−99
CENPA	2p23.3	0.84378996	2.59E−102	6.50E−99
TROAP	12q13.12	0.84327453	4.53E−102	1.01E−98
TPX2	20q11.21	0.84187347	2.05E−101	4.12E−98
KIF4A	Xq13.1	0.84112657	4.57E−101	8.34E−98
CDCA3	12p13.31	0.83985708	1.76E−100	2.95E−97
HJURP	2q37.1	0.83858943	6.70E−100	1.00E−96
NUF2	1q23.3	0.83854781	7.00E−100	1.00E−96
KIFC1	6p21.32	0.83732595	2.51E−99	3.36E−96
PTTG1	5q33.3	0.83712825	3.08E−99	3.87E−96
SKA1	18q21.1	0.83233493	4.15E−97	4.90E−94
CDC25C	5q31.2	0.83159127	8.75E−97	9.76E−94
NEK2	1q32.3	0.82993815	4.54E−96	4.80E−93
SKA3	13q12.11	0.82253139	5.87E−93	5.89E−90

**Table 3:** KEGG pathway enrichment analysis of CDKN3-related genes

Gene set name	Count	p-value	Genes
Cell cycle	41	9.93E−33	E2F1, E2F2, CDC14B, DBF4, PKMYT1, TTK, PTTG2, CHEK1, PTTG1, CCNE2, CCNE1, CDC45, MCM7, CDKN2C, BUB1, ORC6, CCNA2, ORC1, CDC7, CDK1, CDC6, ESPL1, CDC20, MCM2, CDK4, CDC25C, MCM3, MCM4, MCM5, CDK2, CDC25A, MCM6, CDC25B, CCNB1, CCNB2, MAD2L1, PLK1, PCNA, BUB1B, ANAPC7, SMC1B
DNA replication	22	3.25E−24	LIG1, POLA1, POLA2, MCM2, RNASEH2A, MCM3, MCM4, MCM5, MCM6, RFC5, POLD3, PRIM1, DNA2, RFC3, RFC4, MCM7, POLE2, RFC2, POLD1, PRIM2, PCNA, FEN1
Oocyte meiosis	23	2.35E−13	CDK1, CPEB3, PKMYT1, AURKA, PTTG2, CDC20, ESPL1, PTTG1, CDC25C, PPP1CC, CDK2, CCNB1, CCNE2, CCNE1, MAD2L1, CCNB2, SGO1, PLK1, BUB1, FBXO43, FBXO5, ANAPC7, SMC1B
Mismatch repair	11	2.00E−10	EXO1, RFC5, POLD3, MSH6, RFC3, RFC4, RFC2, MSH2, LIG1, POLD1, PCNA
Fanconi anaemia pathway	13	1.65E−08	RAD51C, BLM, EME1, FAAP24, RMI2, BRCA1, RAD51, FANCI, FANCD2, FANCE, FANCG, UBE2T, FANCB
Homologous recombination	10	5.51E−08	POLD3, RAD51C, XRCC3, XRCC2, BLM, POLD1, EME1, RAD54B, RAD54L, RAD51
Progesterone-mediated oocyte maturation	14	3.80E−06	CCNB1, CDK1, MAD2L1, CCNB2, PLK1, CPEB3, BUB1, PKMYT1, ANAPC7, CDC25C, CCNA2, CDC25A, CDK2, CDC25B
Spliceosome	8	7.94E−07	SNRPA1, ALYREF, SNRPD1, SF3A2, SF3B4, RBMX, HNRNPU, SRSF3, HNRNPA3, SRSF9, USP39, SNRPB, SNRPA, SNRPF, THOC1,SNRPG, RBM17

## Discussion

Hepatocellular carcinoma (HCC) is the fifth most common malignant tumour worldwide, and the second deadliest. According to statistical data from the International Centre for Cancer Research [21], the global incidence of HCC has increased more than 626,000 per year, and the fatality rate has reached nearly 600,000 per year. The disease is characterised by rapid development, a high degree of malignancy, a low rate of early diagnosis, and poor prognosis, seriously harming human health and wellbeing. Risk factors for liver cancer include hepatitis B and C infection, cirrhosis, and alcohol intake; 80 to 90% of HCC patients have cirrhosis, which is the most important risk factor [22, 23]. In many liver diseases, there is a significant difference in the incidence of liver cancer between the cirrhotic state and the non-cirrhotic state. The incidence of HCC in cirrhotic patients is significantly higher than that in non-cirrhotic patients, and studies have quantified the correlation between the degree of hepatic fibrosis and liver cancer. Most patients with liver cancer progress to the advanced stage or undergo metastasis, by which time surgical operations struggle to achieve an adequate curative effect, and prognosis is very poor. Sorafenib, the only drug approved by the FDA for advanced liver cancer, can only be extended for 2.8 months [24]. Early screening and preventive treatment for patients with HCC can effectively prolong the 5-year survival rate [25]. However, the mechanism by which cirrhosis develops into liver cancer is still unclear.

Herein, a series of bioinformatics analyses were performed on four independent gene chip databases (from cirrhotic and liver cancer tissue), and 58 common DEGs were identified, of which 12 and 46 were up- and down-regulated, respectively. Among the DEGs, three potential hub genes (CDKN3, CYP2C9 and LCAT) were obtained using the MCODE plug-in of Cytoscape. CDKN3 is a cyclin-dependent kinase inhibitor that interacts with and dephosphorylates Cdk2 kinase to prevent its activation. This gene is deleted, mutated or overexpressed in several cancers [26]. LCAT encodes an extracellular cholesterol esterase, lecithin-cholesterol acyltransferase, and cholesterol esterification is essential for cholesterol transport [25]. CYP2C9 is a C-type member of the cytochrome P450 family 2 subfamily. This monooxygenase catalyses a variety of reactions related to drug metabolism and the synthesis of cholesterol, steroids and other lipids [27–29].

Furthermore, we determined that an up-regulation of CDKN3 is associated with a poor overall survival rate (*p* < 0.05) based on correlations between key genes and clinical data. However, there is controversy over the role of CDKN3 in different tumours; some studies suggest that CDKN3 is a tumour suppressor gene [5], since CDKN3 inhibits cell proliferation in glioblastoma [30], chronic myeloid leukaemia [31], and neuroblastoma [32]. However, the gene is highly expressed in lung cancer [33], nasopharyngeal carcinoma [34], ovarian cancer [35], cervical cancer [36], gastric cancer [37], pancreatic cancer [37], prostate cancer [38] and breast cancer [39], and it promotes cell proliferation and cancer progression. In these cancers, high expression of CDKN3 indicates poor prognosis. In our current study, Oncomine analysis showed that CDKN3 was significantly elevated in anaplastic oligoastrocytoma, leukaemia, HCC, and sarcoma. We demonstrated that CDKN3 was associated with tumour grade, hepatitis virus infection status, microsatellites, and vascular invasion, by verifying the clinical relevance of CDKN3 in patients with HCC. By compared liver cancer, cirrhosis, and normal tissues, CDKN3 was found to be significantly increased in HCC tissues, but the difference was not significant between cirrhosis and normal tissues. This indicates that CDKN3 may be a key gene in the development of HCC in patients with cirrhosis.

In order to explore the mechanism by which CDKN3 influences the occurrence and development of liver cancer, we used bioinformatics tools to screen genes related to changes in CDKN3, and SPC25, CDK1 and CCNB2 were identified. KEGG pathway analysis showed that changes in CDKN3 expression mainly affect pathways related to the cell cycle, DNA replication, meiotic division and mismatch repair, among which most CDKN3-related genes are linked to the cell cycle. However, previous studies have reported contradictory effects of CDKN3 on the cell cycle. Some studies have shown that CDKN3 acts as a cancer-promoting gene in a variety of ways to regulate the G1/S phase transition [40, 41], while others have reported that CDKN3 plays an anti-tumour role via dephosphorylation of CDK2, thereby inhibiting the G1/S phase transition [31]. Therefore, the specific regulatory mechanism by which CDKN3 influences the cell cycle remains unknown. In our current study, LIG1, POLA1, POLA2, MCM2, RNASEH2A and some other genes related to cell cycle regulation were identified. Our results suggest that CDKN3 mainly acts as a tumour-promoting gene by regulating the G1/S phase transition in HCC. Since abnormal expression of genes regulating the cell cycle is closely related to the occurrence and development of tumours, targeted cancer therapy is of great significance. Research on CDKN3 may provide new strategies for the treatment of liver cancer. Therefore, CDKN3 plays a role in the diagnosis, treatment, and prognosis of HCC, and has broad clinical application prospects.

Although previous studies have explored the molecular mechanism by which CDKN3 regulates the conversion from liver cirrhosis to liver cancer [42], only one database study explored differences in CDKN3 between HCC and liver cirrhosis. The relationship between CDKN3 and the occurrence, development, and prognosis of HCC has not been investigated. Other studies based on multiple datasets only focused on screening key genes [43, 44], but did not specifically analyse the molecular mechanism by which core genes play a role.

Through the screening of multiple databases, the present study addressed this issue, by comparing differences between normal tissues, liver cell dysplasia, cirrhosis, HCC, and other pathological states. It is clear that CKDN3 is a key gene affecting the transformation from liver cirrhosis to liver cancer. CKDN3 is closely related to the cell cycle, DNA replication, meiotic division and mismatch repair. Moreover, because CDKN3 is intimately linked to the occurrence, development and prognosis of liver cancer, it may be of value for the early diagnosis and treatment of tumours.

## Conclusion

By integrating multiple microarray gene expression profiles, three key genes (LCAT, CYP2C9, and CDKN3) were identified that appear to be important for the conversion of liver cirrhosis into HCC. Among them, CDKN3 plays a key role in promoting cancer by affecting the cell cycle, which is closely related to the occurrence and development of HCC. Our results illuminate the molecular mechanism by which cirrhosis develops into HCC. The findings may inspire new strategies for screening and monitoring patients with a high risk of cirrhosis, and patients already suffering from HCC.

## Supplementary information

**Additional file 1: Table S1.** The basic clinical info of the patients selected in GSE54236, GSE89377, GSE17548 and GSE63898.

**Additional file 2: Table S2.** Fold changes of the differentially expressed genes shared in GSE54236, GSE89377, GSE17548 and GSE63898.

**Additional file 3: Figure S1.** Association between the expression of LCAT and tumour grade, hepatitis virus infection status, satellites, and vascular invasion in the Wurmbach liver dataset.

**Additional file 4: Figure S2.** Association between the expression of CYP2C9 and tumour grade, hepatitis virus infection status, satellites, and vascular invasion in the Wurmbach liver dataset.

## Data Availability

The datasets during and/or analyzed during the current study available from the corresponding author on reasonable request.

## Abbreviations

HCC: hepatocellular carcinoma
GEO: Gene Expression Omnibus
DEGs: differentially expressed genes
PPI: protein–protein interactions
KEGG: Kyoto Encyclopedia of Genes and Genomes
CDKN3: cyclin-dependent kinase inhibitor 3
CYP2C9: cytochrome P450 family 2 subfamily C member 9
LCAT: lecithin-cholesterol acyltransferase
