# Supplementary figures and images for: Bioinformatics-based screening of key genes for transformation of liver cirrhosis to hepatocellular carcinoma
Source: J Transl Med. 2020 Jan 30;18:40. doi: 10.1186/s12967-020-02229-8 (PMC6993496; doi:10.1186/s12967-020-02229-8)

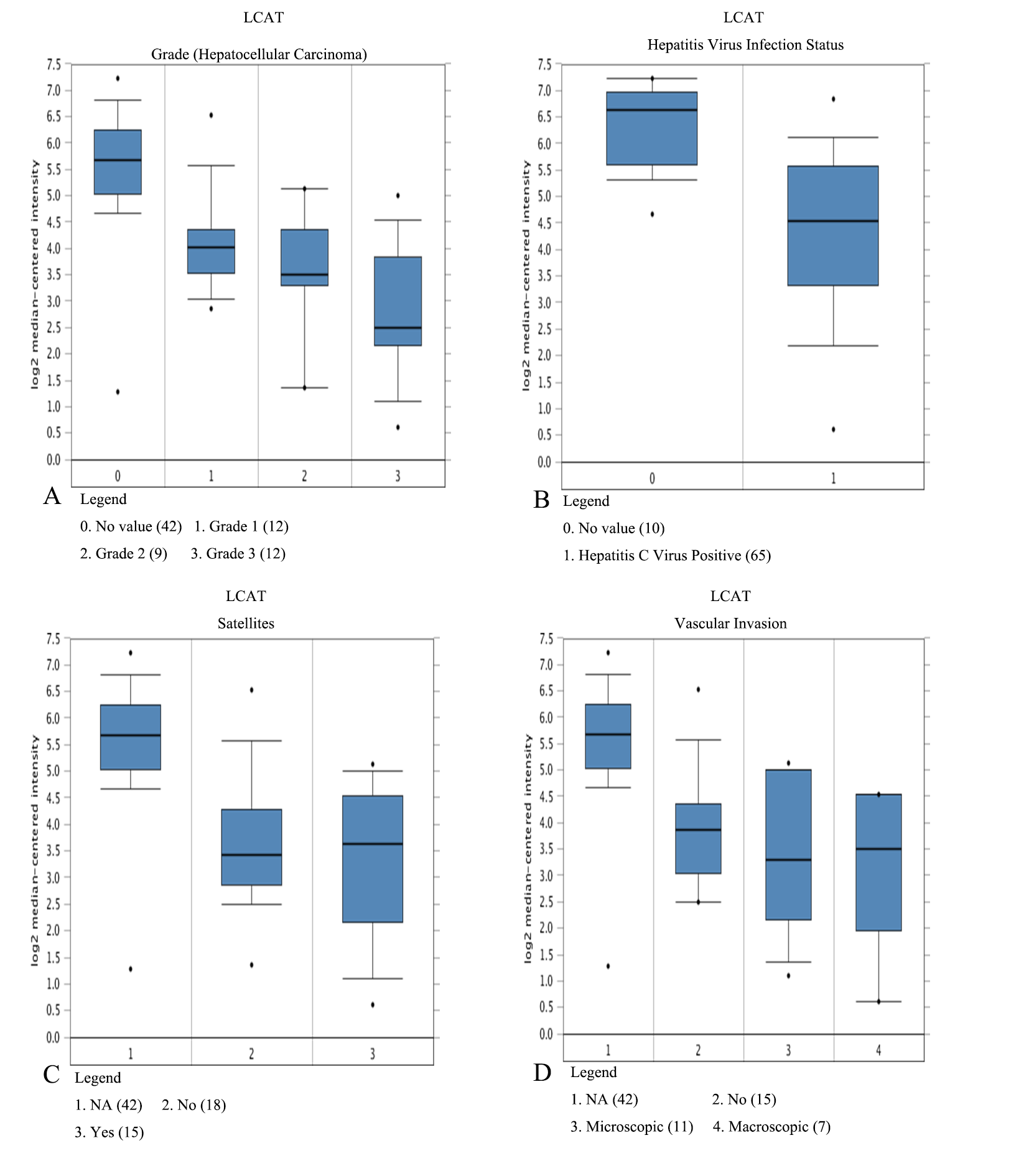

Supplement: Supplementary file 3 — Additional file 3: Figure S1. Association between the expression of LCAT and tumour grade, hepatitis virus infection status, satellites, and vascular invasion in the Wurmbach liver dataset. [file 12967_2020_2229_MOESM3_ESM.tif]

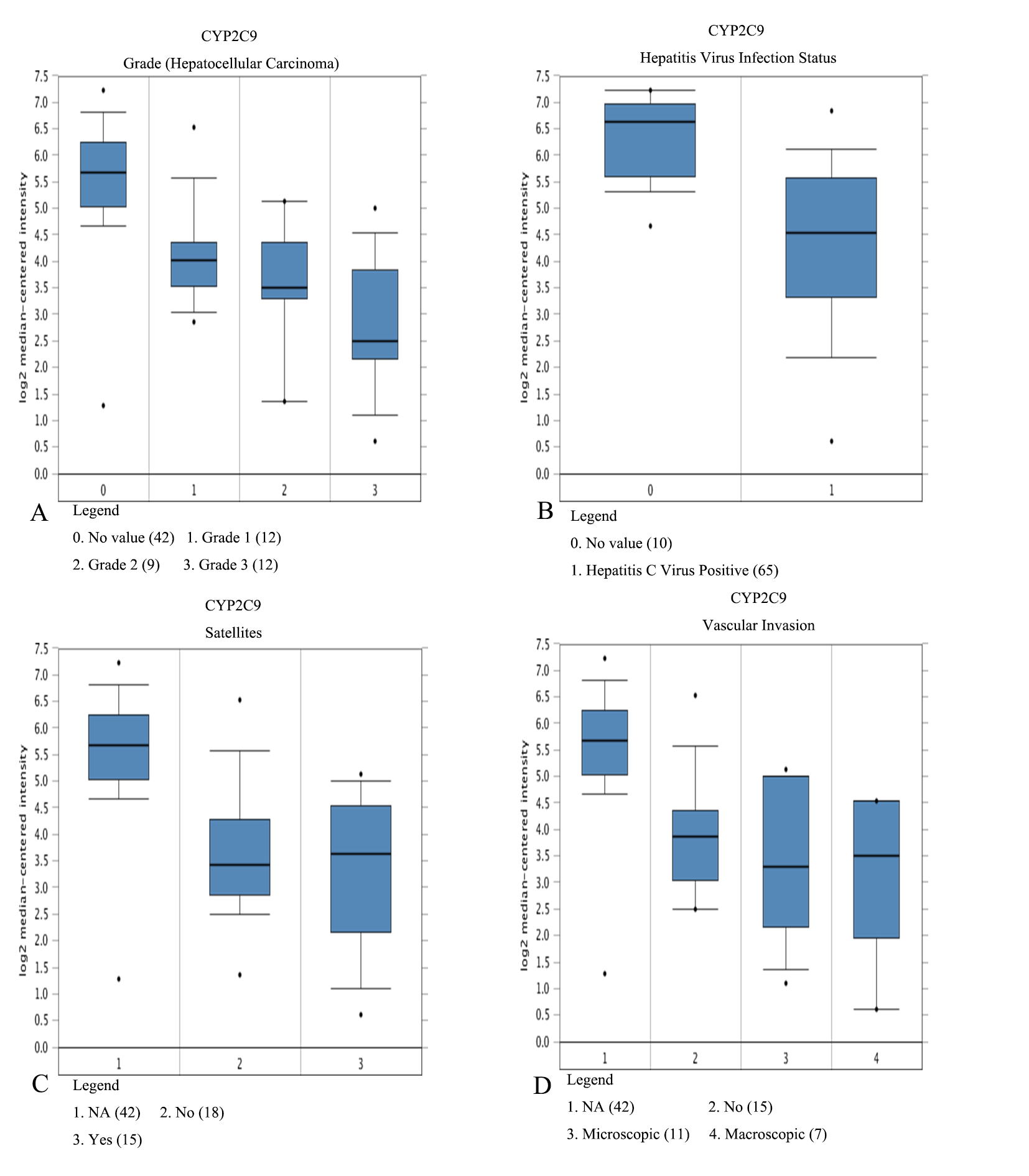

Supplement: Supplementary file 4 — Additional file 4: Figure S2. Association between the expression of CYP2C9 and tumour grade, hepatitis virus infection status, satellites, and vascular invasion in the Wurmbach liver dataset. [file 12967_2020_2229_MOESM4_ESM.png]
